# Corn rootworm (Coleoptera: Chrysomelidae) emergence patterns, survival, and injury in blended refuges and pure stands of short‐stature corn and tall corn

**DOI:** 10.1002/ps.70828

**Published:** 2026-04-20

**Authors:** Devin L. Radosevich, Graham P. Head, Matthew W. Carroll, Aaron J. Gassmann

**Affiliations:** ^1^ Department of Plant Pathology, Entomology and Microbiology Iowa State University Ames IA USA; ^2^ Bayer U.S. – Crop Science Chesterfield MO USA

**Keywords:** *Bacillus thuringiensis*, corn rootworm, insect resistance management, refuge strategy, short‐stature corn, transgenic crops

## Abstract

**BACKGROUND:**

Short‐stature corn became commercially available in the United States in 2025 and has several desirable qualities including lodging resistance and potential for greater planting density. Corn rootworm, *Diabrotica spp.*, are among the most serious insect pests of corn in the United States. However, information about how corn rootworm interact with short‐stature corn is currently lacking. To protect crops from rootworm injury, transgenic corn that produces insecticidal toxins derived from *Bacillus thuringiensis* (Bt) is frequently grown. An important component of insect resistance management for Bt corn is blended refuges, where non‐Bt and Bt plants are planted alongside each other. We conducted a 2‐year field study to quantify patterns of corn rootworm survival, emergence, and injury to short‐stature corn and tall corn across three treatments: pure stand of Bt corn, pure stand of non‐Bt corn, and a blended refuge.

**RESULTS:**

Corn rootworm survival was reduced in Bt corn and in blended refuges, compared to non‐Bt corn. Some differences in survival and timing of emergence were detected between short‐stature corn and tall corn. However, these were not consistent across years or treatments and typically differed between male and female rootworm. Root injury did not differ between tall corn and short‐stature corn across any treatments.

**CONCLUSION:**

Corn rootworm survival, timing of emergence, and injury to corn were generally similar between short‐stature corn and tall corn. This suggests that current insect resistance management strategies for corn rootworm in tall corn are likely appropriate for short‐stature corn, although additional studies are warranted. © 2026 The Author(s). *Pest Management Science* published by John Wiley & Sons Ltd on behalf of Society of Chemical Industry.

## Graphical abstract

Graphical Abstract Text: This study compared corn rootworm (*Diabrotica spp.*) emergence patterns and injury between short‐stature corn and tall corn to help inform corn rootworm management strategies in short‐stature corn

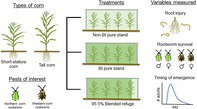

## 1 INTRODUCTION

The corn rootworm complex, *Diabrotica spp*., (Coleoptera: Chrysomelidae) includes western corn rootworm, *Diabrotica virgifera virgifera* LeConte, and northern corn rootworm, *Diabrotica barberi* Smith & Lawrence. These species are among the most economically significant insect pests of corn (*Zea mays* L.) in the United States (US).^1^, ^2^, ^3^, ^4^ Western and northern corn rootworm have one generation per year and overwinter as eggs in the soil, with larvae that hatch in spring and feed on corn roots, their primary food source.^3^, ^5^ Adult rootworm feed on the aboveground tissues of corn plants, such as leaves, pollen, silks and soft kernels.^6^, ^7^ Larvae are the most damaging life stage and are estimated to reduce yield by approximately 15% for every node of roots pruned by larval feeding due in part to a reduction in the capacity of plants to acquire water and nutrients from the soil.^8^, ^9^, ^10^ Furthermore, root feeding by larvae can increase the risk of lodging, which is an inability of a plant to remain upright during the growing season, and this in turn may complicate harvest and further reduce grain yield.^11^, ^12^ In addition to the effects on corn in the US, western corn rootworm also are responsible for economic losses in Europe, where this species has become an invasive pest.^1^, ^9^, ^13^ Currently, corn rootworm are estimated to cost US corn farmers approximately $2 billion annually in expenses related to management costs and yield losses.^14^

To reduce corn rootworm injury, farmers plant transgenic corn that is genetically modified to produce insecticidal toxins derived from the bacterium *Bacillus thuringiensis* (Bt).^15^, ^16^, ^17^, ^18^ Bt corn hybrids targeting corn rootworm larvae were first commercialized in 2003.^19^ However, intense selection pressure imposed through repetitive exposure to Bt toxins for multiple generations has led to wide‐spread, field‐evolved resistance to mCry3A, eCry3.1A, Cry3Bb1 and Gpp34/Tpp35Ab1 in western corn rootworm populations and some instances of Bt resistance in northern corn rootworm.^16^, ^17^, ^20^, ^21^, ^22^, ^23^ This field‐evolved resistance is associated with a genetically based decrease in susceptibility to Bt toxins and higher levels of injury to Bt crops.^18^, ^24^, ^25^

Insect resistance management (IRM) strategies aim to delay the evolution of Bt resistance in pest populations. Two important IRM strategies for Bt crops are non‐Bt refuges and Bt crops that produce a pyramid of Bt toxins.^26^, ^27^ Pyramided Bt crops produce two or more Bt toxins that target the same pest and cause redundant killing where a pest that is resistant to one toxin is killed by another toxin in the pyramid.^15^, ^28^ Refuges involve planting non‐Bt plants in conjunction with Bt plants. These non‐Bt plants (i.e. refuge plants) allow susceptible insects to survive and to then mate with resistant insects emerging from Bt plants, which in turn reduces the proportion of individuals with alleles for resistance to both toxins in a pyramid.^29^ Together, the pyramid and refuge strategies act to delay Bt resistance because individuals that are resistant to one toxin are killed by the other toxin in the pyramid, while refuges act to reduce the number of individuals with genes for resistance to both toxins in the pyramid.^26^, ^30^, ^31^ One method for implementing the refuge strategy involves planting a mixture of Bt and non‐Bt seeds, referred to as a blended refuge, which produces a random arrangement of non‐Bt plants and Bt plants within a field.^32^, ^33^ The US Environmental Protection Agency requires a 5% blended refuge (i.e. 95% Bt plants mixed with 5% non‐Bt plants) for corn with a pyramid of Bt toxins targeting corn rootworm in the US Corn Belt.^34^ Due to limited dispersal of adult rootworm around the time of mating, blended refuges are expected to delay resistance longer than a stand‐alone, or structured, refuge because blended refuges facilitate mating between nearby susceptible and resistant insects.^4^, ^35^, ^36^, ^37^ However, the presence of resistance to current Bt proteins found in corn hybrids in some populations of northern and western corn rootworm underscores the need to reevaluate current IRM approaches, including in light of novel agronomic developments such as short‐stature corn.

Short‐stature corn is a new type of corn already being planted throughout the US Corn Belt. Shorter corn hybrids are being developed by Bayer, Corteva AgriScience, and Stine Seed Company.^38^ Compared to taller hybrids, one noteworthy advantage of short‐stature corn is lodging resistance, which can lead to decreased lodging in short‐stature cornfields.^39^, ^40^ Lodging is a serious issue that may cause yield reductions of 31–43% in corn.^12^, ^41^ Shorter varieties of wheat and rice have been used to improve yields through increased lodging resistance.^42^, ^43^ Short‐stature corn was developed through suppression of gibberellin genes, which lead to decreased length in the internodal spaces.^44^ Short‐stature corn has reduced height and increased stalk circumference but the same number of leaves and nodes as tall‐corn hybrids.^45^ Additionally, because short‐stature hybrids are 61–91 cm shorter, they may better facilitate field access for fertilizer and pesticide applications with ground equipment, particularly in the late growing season when plants are full height.^44^, ^46^ Furthermore, short‐stature corn may be capable of withstanding greater planting densities, an agronomic modification that may in turn increase yield.^47^, ^48^, ^49^ However, more research is necessary to better understand the advantages and disadvantages of short‐stature corn compared to tall corn. In particular, little is currently known about how corn rootworm will interact with short‐stature corn and whether current IRM strategies for tall corn are appropriate for short‐stature corn. To address these knowledge gaps, we designed a field study to test the effects of tall corn and short‐stature corn on patterns of emergence and survival for adult corn rootworm and injury to corn by larval rootworm feeding. These comparisons were made for pure stands of Bt corn, pure stands of non‐Bt corn, and 5% blended refuges. Results of this study will help characterize interactions of corn rootworm with Bt short‐stature corn and inform future IRM research.

## 2 MATERIALS AND METHODS

### 2.1 General experimental approach

This 2‐year study evaluated emergence patterns, survival to adulthood, and injury to corn for naturally occurring populations of western and northern corn rootworm in small plots of pure Bt corn, pure non‐Bt corn, or in a 95%:5% mixture (blended refuge) of Bt and non‐Bt plants, respectively. Mesh cages were installed over each plot so that adult corn rootworm could be collected regularly from inside of cages. Additional plants were grown outside of each cage and used to measure larval rootworm feeding injury.

### 2.2 Experimental design

The experimental design consisted of a randomized complete block design with four blocks, with each block containing two fully crossed factors of corn type and treatment. Corn type was either short‐stature corn or tall corn while treatment was either a pure stand of Bt corn, a pure stand of non‐Bt corn, or a blended refuge of 95% Bt corn and 5% non‐Bt corn. In total, six combinations of factors were evaluated in each block (2 corn types × 3 treatments). The study was repeated over 2 years, in 2022 and 2023. Corn hybrids were provided by Bayer Crop Science (St. Louis, MO) and consisted of SmartStax^®^ hybrids that produce five Bt toxins (Cry1A.105, Cry2Ab2, Cry1F, Cry3Bb1, and Gpp34/Tpp35Ab1) and their associated non‐Bt isolines. Four corn hybrids were used during each year of the study: tall non‐Bt, tall Bt, short‐stature non‐Bt, and short‐stature Bt. For the short‐stature corn used in this study, the short‐stature gene was introduced through conventional breeding. During each year of the study, the tall and short‐stature hybrids were from the same genetic background and differed primarily in the gene that produced the short‐stature phenotype. Hybrids used in 2022 had a 112‐day relative maturity while hybrids planted in 2023 had a 107‐day relative maturity.

Field plots were planted at the Iowa State University Johnson Research Farm in Ames, Iowa in both 2022 and 2023, in an area that was planted with a trap crop for corn rootworm in the previous year. A trap crop contains late‐planted corn (typically planted approximately 4 weeks after commercial planting has ended) and consists of a mixture of maturities that acts to attract adult corn rootworm from the surrounding landscape by providing silks and pollen when these resources are becoming rare in other cornfields.^50^, ^51^ Gravid females are attracted to the trap crop area and will lay eggs in the soil, which then hatch the following year. Thus, the corn rootworm drawn to the trap crop represents a sample of the naturally occurring population from the surrounding area.^51^ Field plots were planted on 20 May in 2022 and on 2 May in 2023. In each year, the study consisted of 24 plots in a 6 × 4 grid that were planted using a tractor (2555 John Deere Tractor) with a 4‐row planter (Max Emerge™ 7100 Integral Rigid Frame Planter; Deere & Company, Moline, IL, USA). Field plots were four rows wide and 6.1 m long with 0.76 m between rows and 15.2 cm between seeds in a row, leading to a planting density of 86 487 plants per hectare (35 000 plants per acre). There was 3 m of fallow ground separating field plots. Plots with pure stands of Bt or non‐Bt corn were planted only with a tractor while blended refuge plots were first planted to a pure stand of Bt corn with a tractor, but the furrows were left open. Five Bt seeds in the first 4 m of the rows and two Bt seeds in the last 1.5 m of the rows were then removed and replaced with non‐Bt seeds. Furrows were covered with soil and positions of the non‐Bt seeds marked with flags. The five non‐Bt seeds in the first 4 m of a row represented a 5% refuge over which cages were later installed (approximately 96 plants per cage), and the two non‐Bt seeds in the last 1.5 m of a row represented a 5% refuge for the plants left outside of the cages (approximately 40 plants), which were sampled to measure root injury.

In the third week of June, before corn rootworm adults began to emerge, steel frames (3.8 m long × 3.2 m wide × 2.4 m tall) were positioned over each plot, and heavy‐duty mesh cages were hung over the steel frames (Redwood Empire Awning, Santa Rosa, CA, USA). The bottoms of mesh cages were buried in the soil to prevent escape of adult corn rootworm. All plants were trimmed to approximately 60 cm in height in early July (2022) and late June (2023) to assist in the capture of adult corn rootworm inside mesh cages.

Beginning on June 29, 2022 and June 26, 2023, cages were carefully inspected and all adult corn rootworm were collected from inside the cages three times each week, approximately every other day, using 50 mL centrifuge tubes (CentriStar™, Corning Inc., Corning, NY, USA). Corn rootworm adults were manually collected with mouth aspirators fitted to the lid of the centrifuge tube (product #1135A, BioQuip, Rancho Dominguez, CA, USA). After collecting adults, 50 mL centrifuge tubes with rootworm adults were placed in a −20 °C freezer for at least 1 week, with adult rootworm subsequently placed in a solution of 85% ethanol. Adults were separated into western, northern, and southern corn rootworm (*Diabrotica undecimpunctata howardi* Barber), according to a dichotomous key.^52^ Sex of adults was determined for each individual by observing the morphology of the basitarsal pad through a microscope (Leica MZ6, Leica Microsystems, Wetzlar, Germany) following Hammack and French.^53^ Field experiments were terminated on 30 September 2022 and on 18 September 2023 after the cages yielded zero western and northern corn rootworm adults for two consecutive collection periods.

Roots from plants grown immediately outside of the cages were used to evaluate larval rootworm feeding injury based on a 0–3 node‐injury scale.^54^ Roots were dug from plots on 4 August in 2022 and on 21 July in 2023 and washed to remove soil. Five to six roots were dug per plot from each pure stand plot of Bt and non‐Bt corn, and five to six Bt roots and two non‐Bt roots were dug from each blended refuge plot. Plants in the blended refuge plots were tested for the presence of Bt toxins using an ELISA‐based kit (QuickStix combo strips for Cry2A/3B/1F/34; EnviroLogix, Portland, ME) before scoring injury.

### 2.3 Data analysis

Data analyses were carried out using SAS version 9.4 (SAS Institute, Cary, NC, USA), and data were analyzed separately for each year of the study. A mixed‐model analysis of variance (ANOVA) was used to test for the effects of corn type, treatment, and their interaction with the number of adult corn rootworm collected (i.e. survival to adulthood), the timing of adult emergence, and root injury (PROC GLIMMIX). For analysis of survival and timing of adult emergence, the fixed factors in the analysis were corn type (short‐stature or tall), treatment (Bt pure stand, non‐Bt pure stand, or blended refuge), sex (male or female), and all possible interactions among these factors. Random factors in the analyses were block and the interaction of block with all fixed factors. Survival of western and northern corn rootworm adults per plot was standardized to the number of adults per plant to account for variability in the number of plants in each plot. This was accomplished by dividing the total number of either male or female adults that emerged in a plot by the number of corn plants in that plot. In 2022, the number of plants per plot ranged from 94 to 105 (98.9 ± 3.2 (mean ± SD)). During 2023, plots had 91 to 124 plants per plot (105.9 ± 7.9). Prior to analysis, the number of adults per plant was transformed with a square‐root function to improve for normality of residuals and homogeneity of variance. The date of collection for each individual corn rootworm adult was converted to Julian day (1 January = day 1 and 31 December = day 365). Data on the timing of adult emergence (Julian day) was transformed by the natural log function prior to analysis. When there were significant effects, pairwise comparisons were conducted to separate significantly different means based on Tukey's honestly significant difference (HSD) with α = 0.05 (PDIFF in PROC GLIMMIX).

Data on root injury were analyzed with an ANOVA that included the fixed factors of corn type (short‐stature or tall), treatment (Bt pure stand, non‐Bt pure stand, or blended refuge), Bt trait (Bt corn or non‐Bt corn), and the interactions of corn type with treatment and corn type with Bt trait nested within treatment, with Bt trait coded as a nested factor within treatment because the blended refuge had both Bt and non‐Bt plants. Random factors were block and all of the interactions of block with the fixed factors. To ensure normality of residuals and homogeneity of variance, data were transformed with a square‐root function. Pairwise comparisons were made using Tukey's HSD with α = 0.05 (PDIFF in PROC GLIMMIX).

## 3 RESULTS

### 3.1 Survival to adulthood

There were 26,530 western corn rootworm adults collected in this study: 15,779 in 2022 and 10,751 in 2023. Additionally, 2,389 northern corn rootworm adults were collected: 832 in 2022 and 1,557 in 2023. Some southern corn rootworm (1,829 in total) were collected, but this species was not included in the data analysis because it is not considered an economically important pest of corn in the US Corn Belt.^55^ As such, approximately 91% of the adults included in the analyses were western corn rootworm and 9% were northern corn rootworm.

**Figure 1:**
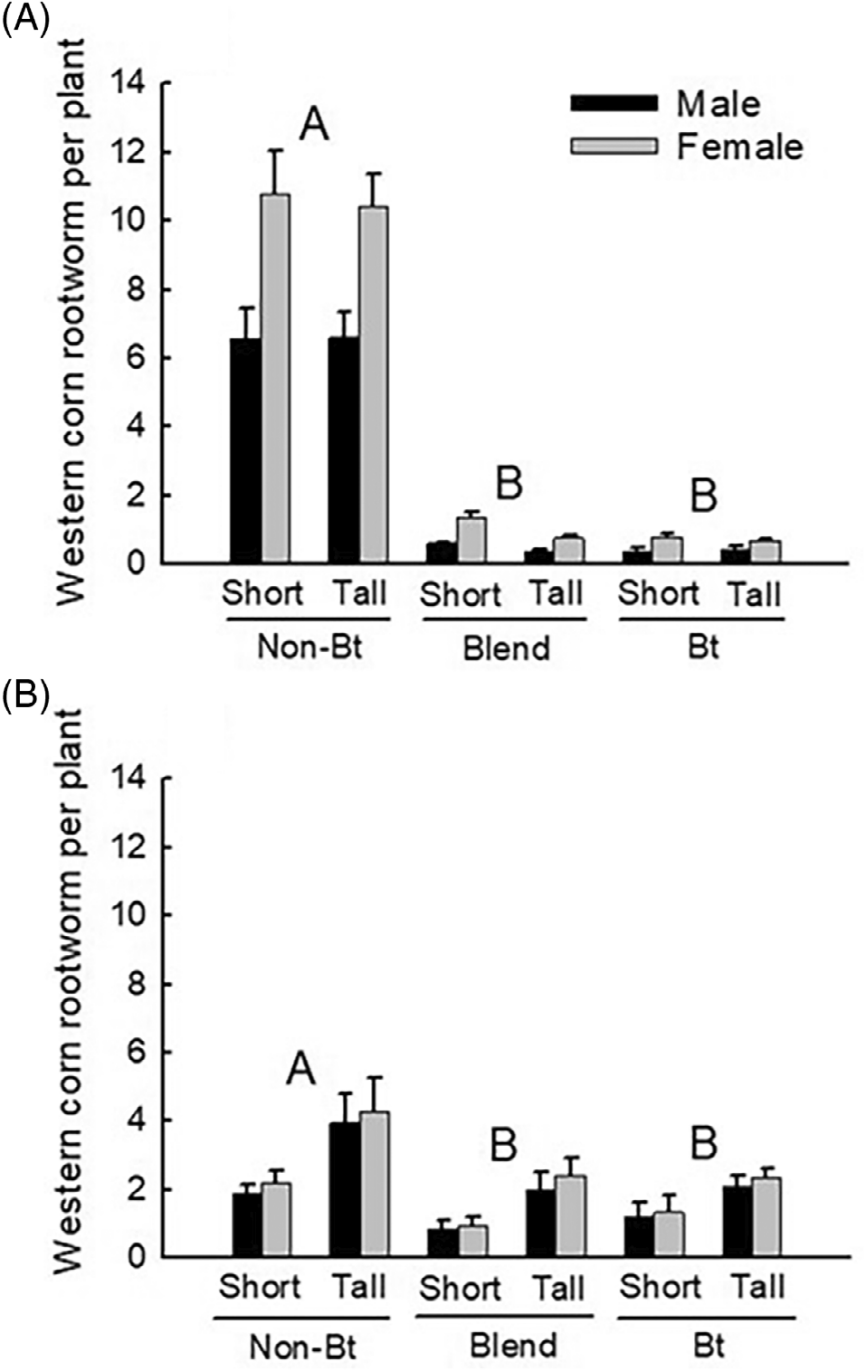
Survival of western corn rootworm (*Diabrotica v. virgifera*) adults per plant in field plots during (A) 2022 and (B) 2023. The *x*‐axis displays short‐stature corn (Short) and tall corn (Tall) across the treatments: non‐Bt pure stand (Non‐Bt), blended refuge (Blend), and Bt pure stand (Bt). Letters indicate significant differences between treatments. In both years, significantly more females emerged than males. Bar heights are means and error bars are the standard error of the mean.

Survival of western corn rootworm in 2022 was similar between short‐stature corn and tall corn (Table 1, Fig. 1(A)). However, there was a marginally significant difference between short and tall corn in 2023, with greater emergence of western corn rootworm from tall corn compared to short‐stature corn (Table 1, Fig. 1(B)). Additionally, survival differed among treatments in both years. In 2022 and 2023, significantly more adult western corn rootworm emerged from the non‐Bt pure stand than the blended refuge or Bt pure stand, and the number of adults was similar between the blended refuge and Bt pure stand (Fig. 1). Significantly more female than male western corn rootworm emerged in both years of the study, but in 2022, there was also a significant interaction between treatment and sex, with a more pronounced difference between females and males in the non‐Bt pure stand than the other treatments (Table 1, Fig. 1).

**Table 1:** Analysis of variance for survival to adulthood of western corn rootworm *Diabrotica v. virgifera*) in 2022 and 2023

Source^†^	*df*	*F* value	*P*
2022			
Corn	1,3	1.03	0.3850
Treatment	2,6	311	<0.0001
Sex	1,3	174	0.0009
Corn × Treatment	2,6	0.76	0.5061
Corn × Sex	1,3	2.60	0.2050
Treatment × Sex	2,6	103	<0.0001
Corn × Treatment × Sex	2,6	0.82	0.4846
2023			
Corn	1,3	9.45	0.0544
Treatment	2,6	5.64	0.0418
Sex	1,3	15.1	0.0301
Corn × Treatment	2,6	0.11	0.9009
Corn × Sex	1,3	1.29	0.2863
Treatment × Sex	2,6	0.25	0.7845
Corn × Treatment × Sex	2,6	1.50	0.2730

^†^ Fixed factors in the analysis were: Corn (short‐stature or tall), Treatment refers to non‐Bt pure stand, Bt pure stand, or a 5% blended refuge, and Sex is the sex of the adult rootworm (male or female).

**Figure 2:**
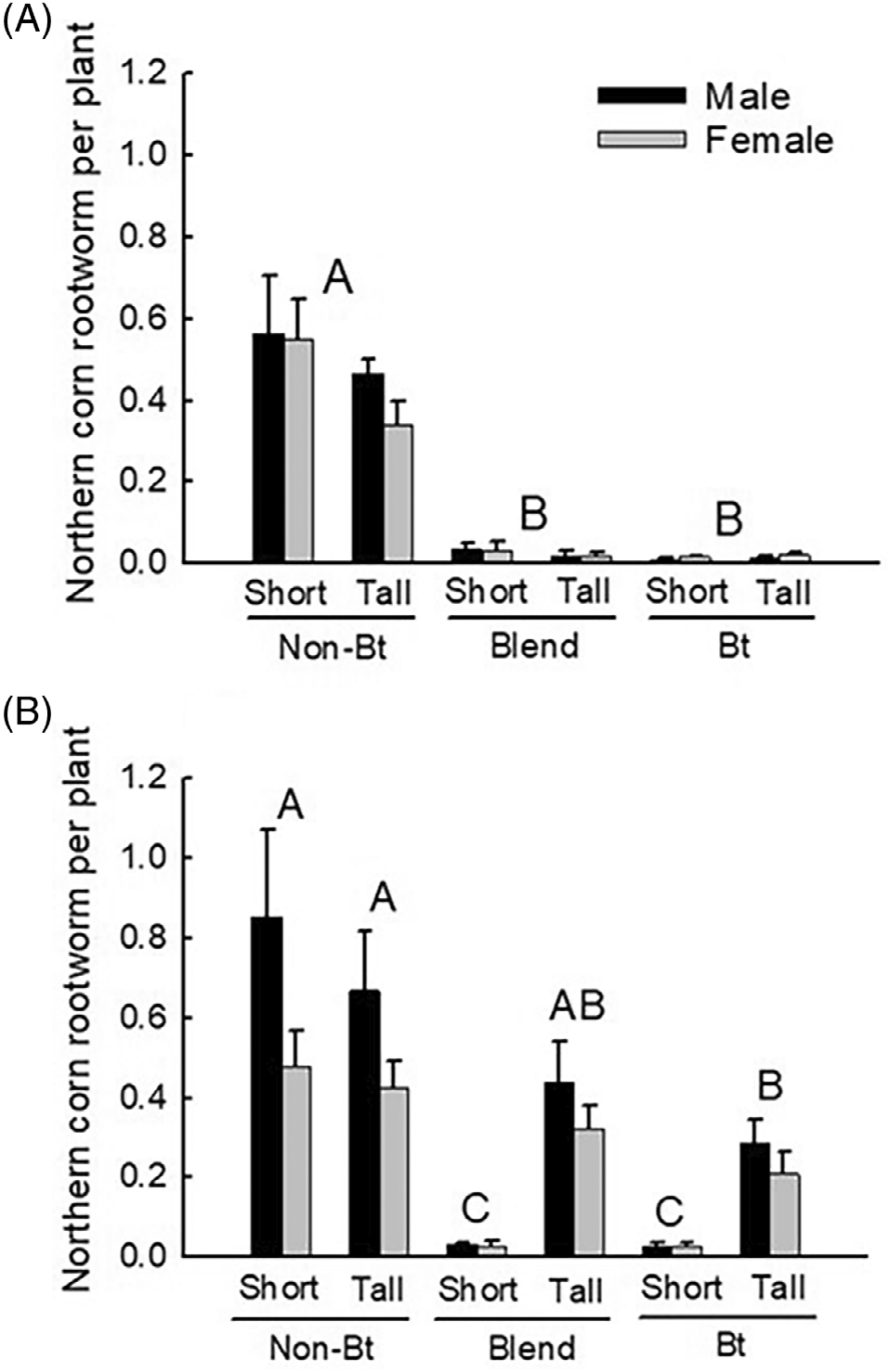
Survival of northern corn rootworm (*Diabrotica barberi*) adults per plant in field plots during (A) 2022 and (B) 2023. The *x*‐axis displays short‐stature corn (Short) and tall corn (Tall) across the treatments: non‐Bt pure stand (Non‐Bt), blended refuge (Blend), and Bt pure stand (Bt). Letters indicate significant differences between treatments in 2022 and among combinations of treatment and corn type (i.e. short‐stature corn and tall corn) in 2023. In addition, significantly more males emerged than females in 2023. Bar heights are means and error bars are the standard error of the mean.

In 2022, only treatment had a significant effect on survival for northern corn rootworm. Significantly more adults emerged from the non‐Bt pure stand than the Bt pure stand or blended refuge, and survival did not differ between the blended refuge and Bt pure stand (Table 2, Fig. 2(A)). During 2023, a significant interaction was present between corn type and treatment (Table 2). Significantly more northern corn rootworm emerged from tall corn than short‐stature corn in the blended refuge and Bt pure stand, but survival in the non‐Bt pure stand was similar between short‐stature corn and tall corn (Fig. 2(B)). Survival on short‐stature corn was significantly greater in the non‐Bt pure stand than the blended refuge or Bt pure stand, which did not differ from each other (Fig. 2(B)). By contrast, on tall corn, survival was greater in the non‐Bt pure stand compared to the Bt pure stand, but the blended refuge did not significantly differ from either of these treatments (Fig. 2(B)). Additionally, there was a significant effect of sex with significantly more males emerging than females overall in 2023.

**Table 2:** Analysis of variance for survival to adulthood of northern corn rootworm (*Diabrotica barberi*) in 2022 and 2023

Source^†^	*df*	*F* value	*P*
*2022*			
Corn	1,3	1.12	0.3678
Treatment	2,6	96.4	<0.0001
Sex	1,3	0.10	0.7699
Corn × Treatment	2,6	1.27	0.3472
Corn × Sex	1,3	0.22	0.6694
Treatment × Sex	2,6	2.46	0.1658
Corn × Treatment × Sex	2,6	1.09	0.3945
*2023*			
Corn	1,3	18.7	0.0228
Treatment	2,6	40.7	0.0003
Sex	1,3	15.1	0.0303
Corn × Treatment	2,6	12.9	0.0067
Corn × Sex	1,3	0.05	0.8328
Treatment × Sex	2,6	4.82	0.0565
Corn × Treatment × Sex	2,6	0.98	0.4289

^†^ Fixed factors in the analysis were: Corn (short‐stature or tall), Treatment refers to non‐Bt pure stand, Bt pure stand, or a 5% blended refuge, and Sex is the sex of the adult rootworm (male or female).

### 3.2 Timing of emergence

**Figure 3:**
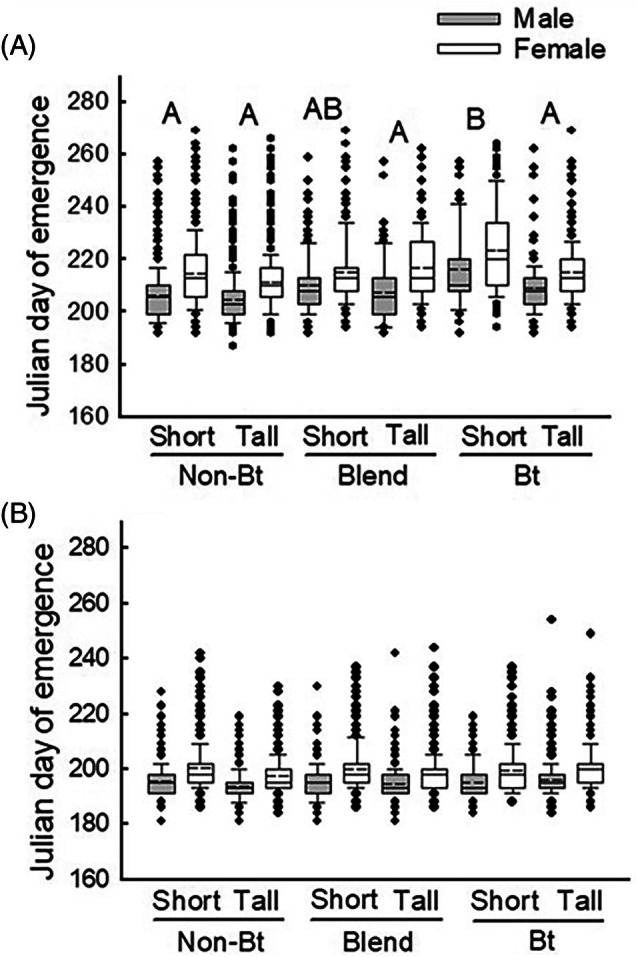
Timing of emergence for adult western corn rootworm (*Diabrotica v. virgifera*) from the field plots in (A) 2022 and (B) 2023. The *x*‐axis denotes short‐stature corn (Short) and tall corn (Tall) across the treatments: non‐Bt pure stand (Non‐Bt), blended refuge (Blend), and Bt pure stand (Bt). Boxes represent the 75^th^ to 25^th^ percentiles while the whiskers on boxes are the 95^th^ and 5^th^ percentiles. Points outside of the whiskers are data points outside the 5^th^ and 95^th^ percentiles. In the boxes, solid lines represent the median and dashed lines are the mean. For 2022, letters indicate significant differences between combinations of corn type (i.e. short or tall) and treatment. In both 2022 and 2023, males emerged significantly earlier than females.

Timing of emergence for western corn rootworm adults was affected by a significant three‐way interaction among corn type, treatment, and sex in 2022. In 2023, there was a significant interaction between corn type and treatment and a significant effect of sex (Table 3). During 2022, western corn rootworm emergence was significantly delayed on short‐stature corn compared to tall corn in the Bt pure stand, but short‐stature corn and tall corn did not differ in the non‐Bt pure stand or blended refuge (Fig. 3(A)). While males emerged before females, the magnitude of this difference varied among combinations of treatment and corn type. Despite the significant interaction between corn type and treatment in 2023, Tukey's HSD did not identify any significant differences among combinations of corn type and treatment (Table 3, Fig. 3(B)). As with the previous year, males emerged significantly earlier than females in 2023 (Table 3).

**Table 3:** Analysis of variance for timing of emergence for western corn rootworm (*Diabrotica v. virgifera*) adults in 2022 and 2023

Source^†^	*df*	*F* value	*P*
2022			
Corn	1,3	27.2	0.0137
Treatment	2,6	11.2	0.0094
Sex	1,3	403	0.0003
Corn × Treatment	2,6	7.55	0.0230
Corn × Sex	1,3	0.76	0.4479
Treatment × Sex	2,6	0.74	0.5147
Corn × Treatment × Sex	2,6	11.1	0.0097
2023			
Corn	1,3	3.26	0.1688
Treatment	2,6	0.64	0.5577
Sex	1,3	880	<0.0001
Corn × Treatment	2,6	6.94	0.0275
Corn × Sex	1,3	9.79	0.0521
Treatment × Sex	2,6	0.27	0.7687
Corn × Treatment × Sex	2,6	0.58	0.5895

^†^ Fixed factors in the analysis were: Corn (short‐stature or tall), Treatment refers to non‐Bt pure stand, Bt pure stand, or a 5% blended refuge, and Sex is the sex of the adult rootworm (male or female).

**Figure 4:**
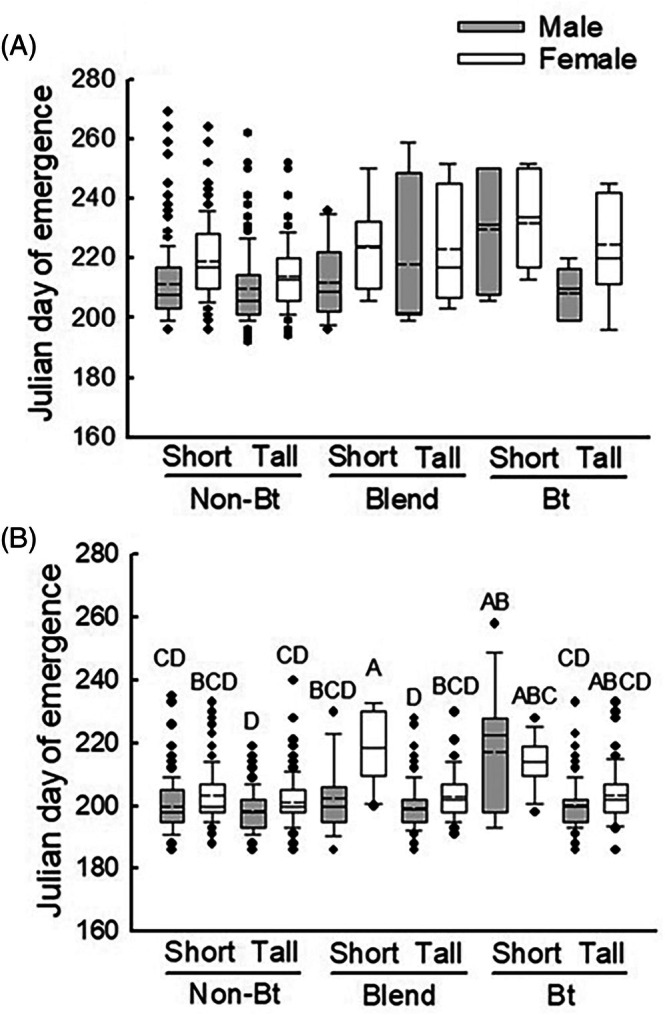
Timing of emergence for adult northern corn rootworm (*Diabrotica barberi*) from the field plots in (A) 2022 and (B) 2023. The *x*‐axis denotes short‐stature corn (Short) and tall corn (Tall) across the treatments: non‐Bt pure stand (Non‐Bt), blended refuge (Blend), and Bt pure stand (Bt). Boxes represent the 75^th^ to 25^th^ percentiles while the whiskers on boxes are the 95^th^ and 5^th^ percentiles. Points outside of the whiskers lie outside the 5^th^ through 95^th^ percentile window. In the boxes, solid lines represent the median and dashed lines are the mean. In 2022, males emerged significantly earlier than females. For 2023, letters indicate significant differences between means.

In 2022, the timing of northern corn rootworm adult emergence from short‐stature corn resembled that of tall corn. The only significant effect on timing of adult emergence was sex with males emerging earlier than females (Table 4, Fig. 4(A)). There was a significant three‐way interaction among corn type, treatment, and sex for northern corn rootworm in 2023 (Table 4). In 2023, female emergence in the blended refuge was significantly delayed on short‐stature corn compared to tall corn, and males in the Bt pure stand emerged significantly later from short‐stature corn than tall corn (Fig. 4(B)).

**Table 4:** Analysis of variance for timing of emergence for northern corn rootworm (*Diabrotica barberi*) adults in 2022 and 2023

Source^†^	*df*	*F* value	*P*
2022			
Corn	1,3	7.86	0.0677
Treatment	2,6	2.55	0.1577
Sex	1,3	12.3	0.0391
Corn × Treatment	2,6	2.84	0.1357
Corn × Sex	1,3	0.45	0.5226
Treatment × Sex	2,6	0.33	0.7299
Corn × Treatment × Sex	2,6	2.28	0.1649
2023			
Corn	1,3	81.4	0.0029
Treatment	2,6	26.6	0.0049
Sex	1,3	31.5	0.0112
Corn × Treatment	2,6	17.4	0.0107
Corn × Sex	1,3	2.18	0.2365
Treatment × Sex	2,6	10.0	0.0278
Corn × Treatment × Sex	2,6	8.78	0.0344

^†^ Fixed factors in the analysis were: Corn (short‐stature or tall), Treatment refers to non‐Bt pure stand, Bt pure stand, or a 5% blended refuge, and Sex is the sex of the adult rootworm (male or female).

### 3.3 Root injury

**Figure 5:**
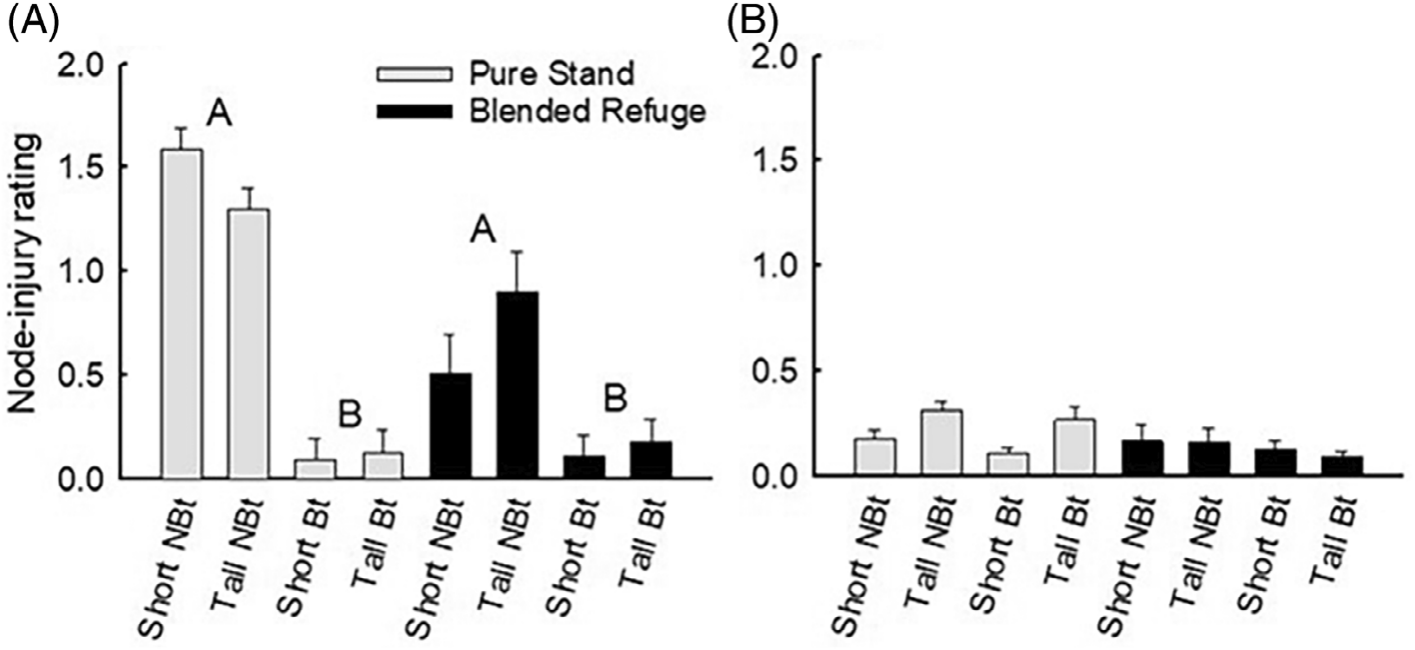
Corn rootworm larval feeding injury in (A) 2022 and (B) 2023. Labels on the *x*‐axis display corn type, short‐stature corn (Short) or tall corn (Tall), and Bt trait (NBt = non‐Bt and Bt = Bt). Letters in 2022 indicate significant differences between Bt corn and non‐Bt corn in the three treatments: non‐Bt pure stand, Bt pure stand, and blended refuge. Bar heights are sample means and error bars are the standard error of the mean.

Corn rootworm injury did not differ significantly between short‐stature corn and tall corn in either year of the study. For root injury in 2022, there were significant effects of treatment and Bt trait nested within treatment. Non‐Bt plants experienced significantly more feeding injury from corn rootworm larvae than Bt plants, regardless of treatment (Table 5; Fig. 5(A)). Injury ratings for non‐Bt plants from the blended refuge were lower but not significantly different from non‐Bt plants in the non‐Bt pure stand. The node‐injury ratings were substantially lower in 2023 compared to 2022, and none of the effects were significant in 2023 (Table 5; Fig. 5(B)).

**Table 5:** Analysis of variance for node‐injury ratings in 2022 and 2023

Source^†^	*df*	*F* value	*P*
2022			
Corn	1,3	0.08	0.8001
Treatment	2,3	51.9	0.0047
Bt trait [Treatment]	1,3	27.4	0.0136
Corn × Treatment	2,9	3.21	0.0885
Corn × Bt trait [Treatment]	1,9	0.57	0.4708
2023			
Corn	1,3	2.30	0.2270
Treatment	2,3	3.73	0.1537
Bt trait [Treatment]	1,3	0.84	0.4275
Corn × Treatment	2,9	2.04	0.1856
Corn × Bt trait [Treatment]	1,9	0.13	0.7261

^†^ Fixed factors in the analysis were: Corn (short‐stature or tall), Treatment refers to non‐Bt pure stand, Bt pure stand, or a 5% blended refuge, and Bt trait represents Bt expression of the sampled plant (either non‐Bt or Bt toxin producing). Bt trait is nested within treatment because the blended refuge had Bt corn and non‐Bt corn.

## 4 DISCUSSION

Before this study, little was known about how corn rootworm will interact with short‐stature corn. We found that corn rootworm survival, emergence patterns, and injury on short‐stature corn largely resembled those on tall corn among the Bt pure stand, non‐Bt pure stand, and blended refuge. Differences between short‐stature corn and tall corn were not consistent between years and may have arisen from a variety of factors, including the genetic background of the corn hybrids studied. In our study, survival to adulthood was generally similar between short‐stature corn and tall corn, suggesting that the production of refuge individuals from non‐Bt corn, relative to survival on Bt corn, would be similar between short‐stature and tall corn. Similar studies found that significantly more corn rootworm survived in a non‐Bt pure stand compared to a Bt pure stand.^20^, ^37^, ^51^, ^56^, ^57^ Furthermore, previous studies have shown that male corn rootworm emerge significantly earlier, approximately 6 days sooner, than females.^20^, ^37^, ^51^, ^56^ These results are consistent with our results for both western and northern corn rootworm. Additionally, root‐injury ratings from short‐stature corn resembled those from tall corn, hence Bt traits seemed to provide equal protection against larval rootworm feeding in short‐stature and tall corn. Since interactions with corn rootworm appear similar between short‐stature corn and tall corn, the results of this study suggest that current IRM strategies for tall corn should be appropriate for short‐stature corn. However, additional research on patterns of pest movement and mating in blended refuges of short‐stature corn should be conducted to provide a more complete understanding of the value of blended refuges to delay Bt resistance in short‐stature corn.^36^, ^37^, ^58^

Compared to pure stands of Bt or non‐Bt corn, blended refuges can affect certain variables including rootworm survival, timing of adult emergence, and root injury.^4^, ^37^, ^51^ Overall in this study, patterns of root injury and rootworm survival were similar between short‐stature corn and tall corn in the blended refuge. Previous studies found similar corn rootworm survival between the blended refuge and Bt pure stand while survival was greatest in the non‐Bt pure stand.^20^, ^37^, ^51^, ^56^ Similarly, in our study, survival in the Bt pure stand resembled the blended refuge with greater survival in the non‐Bt pure stand, for both short‐stature corn and tall corn (Figs 1 and 2). Additionally, injury ratings on non‐Bt plants tended to be lower in the blended refuge than in the non‐Bt pure stand, although this was not statistically significant (Fig. 5). The observation of lower root injury for non‐Bt plants in a blended refuge likely arises because of reduced movement of larvae from neighboring Bt plants, which kill rootworm larvae, compared to a non‐Bt pure stand where adjacent plants are non‐Bt and support a high abundance of larvae.^4^, ^45^, ^59^

Delays in adult emergence for females from Bt corn in a blended refuge have important implications for IRM. Specifically, although male corn rootworm typically emerge before females, greater asynchronous emergence between susceptible males from refuge plants and females from Bt plants can lead to assortative mating, which may accelerate resistance evolution.^60^, ^61^ For example, females from Bt corn have been observed emerging 9–12 days later than males from non‐Bt corn within a blended refuge.^37^, ^56^ In the short‐stature corn blended refuge, northern corn rootworm males emerged approximately 18 days before females, although it is not known to what extent these individuals were from Bt *versus* non‐Bt plants (Fig. 4(B)). In general, the closer spatial proximity of Bt and non‐Bt plants in a blended refuge should facilitate mating between refuge individuals and insects emerging from Bt plants, helping to reduce the negative effects of limited adult dispersal and temporal asynchrony in emergence from Bt and non‐Bt plans. However, these benefits arise at the cost of sublethal exposure to Bt toxins when larvae move between Bt and non‐Bt plants.^4^, ^26^, ^36^, ^37^

In the current study, despite collecting nearly 11 times more western corn rootworm than northern corn rootworm, survival and emergence patterns were similar in most instances between rootworm species and short‐stature corn and tall corn. For both rootworm species, survival was significantly greater in the non‐Bt pure stand than the Bt pure stand (Figs 1 and 2). In addition, emergence time did not differ between short‐stature corn and tall corn in one of two years (Figs 3(B) and 4(A)). In our study, significant differences in survival or delays in emergence between short‐stature corn and tall corn only occurred in treatments that had Bt plants. For example, significantly more northern corn rootworm emerged on tall corn compared to short‐stature corn in the Bt pure stand and blended refuge (Fig. 2(B)). Further, western and northern corn rootworm experienced significant delays in emergence on short‐stature corn compared to tall corn in the Bt pure stand and blended refuge (Figs. 3(A) and 4(B)). These effects may have arisen because of morphological differences between short‐stature corn and tall corn. However, additional research would be necessary to understand why such differences arose between short‐stature corn and tall corn in the blended refuge and Bt pure stand.

Delayed emergence and reduced survival on Bt short‐stature corn compared to Bt tall corn could alter resistance allele frequency and mating between susceptible insects and resistant insects.^26^, ^60^, ^62^ As mentioned previously, delayed emergence from Bt corn compared to non‐Bt corn could lead to assortative mating between Bt‐selected individuals and refuge individuals, thus accelerating resistance evolution. By contrast, decreased emergence of adults from Bt corn could increase the effective size of the refuge population, acting to delay resistance evolution.^63^ Further research would be needed to understand the mechanisms driving the differences in survival and emergence observed here between short‐stature corn and tall corn, which may have arisen from factors related to nutritional quality of the host plants, physical attributes of the roots, or other factors.

Corn rootworm populations in the current study likely possessed some degree of resistance to Cry3Bb1 and Gpp34/Tpp35Ab1 based on previous work identifying Bt resistance in Iowa, including populations that were collected near the location of our study.^21^, ^64^ To the extent that corn rootworm developing on Bt corn were resistant to the Bt toxins present, survival to adulthood and root injury could become more similar to non‐Bt corn.^24^, ^65^ In 2022, root injury and rootworm survival were significantly greater in the non‐Bt pure stand compared to the Bt pure stand (Fig. 5(A)), suggesting that the corn rootworm encountered had some degree of susceptibility to Bt. In 2023, injury did not differ between Bt plants and non‐Bt plants across any of the treatments. However, feeding injury was much lower that year, which could explain the lack of difference among treatments. While the corn rootworm in this study likely possessed some level of Bt resistance, lower survival in the Bt pure stand relative to the non‐Bt pure stand suggests that resistance was incomplete or there was a mix of susceptible and resistant individuals.^66^ Past studies have shown that corn rootworm developing on Bt corn are delayed 4–8 days compared to those on non‐Bt corn.^20^, ^51^, ^57^, ^65^ In our study, emergence from the Bt pure stand was rarely significantly delayed compared to the non‐Bt pure stand, an effect that may be due to the presence of Bt resistance genes in rootworm surviving to adulthood on Bt corn.^21^ Bioassays focusing on corn rootworm survival on Bt plants would be needed to evaluate the degree of Bt resistance present in the populations observed in our study.

Field‐evolved Bt resistance in western and northern corn rootworm negatively impacts farmers, and short‐stature corn likely will incorporate Bt toxins for which some rootworm populations have already evolved resistance. Resistance to Cry3Bb1 and Gpp34/Tpp35Ab1 by western and northern corn rootworm currently exists in the US Corn Belt,^17^, ^21^, ^22^ and resistance is becoming more widespread.^21^, ^64^, ^67^, ^68^, ^69^, ^70^, ^71^ Based on our results, the outcomes of resistance management in tall corn are not expected to differ for short‐stature corn. To help combat the continued development of resistance in corn rootworm, Bt pyramids are recommended because individuals that are resistant to one toxin may be killed by the other toxin present in the pyramid.^26^, ^28^ However, resistance to one Bt toxin present in a pyramid could effectively turn the pyramid into a single‐toxin crop, with the benefit of combining toxins becoming reduced or lost.^63^ Farmers can further delay and mitigate resistance in corn rootworm by planting adequate refuges and using alternative management strategies.^67^, ^72^ Alternative strategies include rotating to a non‐host crop, such as soybean, or rotating Bt corn with non‐Bt corn combined with soil‐applied insecticides.^65^, ^73^, ^74^

Additional studies can help to refine IRM strategies for corn rootworm in short‐stature corn. For example, corn rootworm strains with known levels of resistance to Cry3Bb1 and Gpp34/Tpp35Ab1 could be used to compare survival and emergence on Bt short‐stature corn and Bt tall corn. Future research on short‐stature corn should include measurements of emergence and mating rates between corn rootworm adults from Bt *vs*. non‐Bt plants in a blended refuge, similar to Taylor and Krupke.^36^ Finally, other shorter‐stature corn hybrids, developed through either genetic engineering or conventional breeding, may differ phenotypically from the hybrids used in this study.^38^ These phenotypic differences could lead to differences in corn rootworm interactions among short‐stature corn hybrids. Future research could determine how corn rootworm interactions compare among these hybrids of short‐stature corn.

One possible benefit of short‐stature corn is its tolerance of greater planting density, which may increase yield by allowing more plants to be grown per area of land.^47^, ^48^ Tolerance of greater planting density in short‐stature corn may be associated with a more upright leaf angle, which decreases shading and increases light interception of the canopy, and with improved remobilization of biomass from the stem during the reproductive stages.^49^, ^75^, ^76^ Greater planting density (i.e. 38 cm row spacing compared to the 76 cm row spacing used in our study) has been found to increase the number of western and northern corn rootworm adults that emerge per m^2^ of soil, but larval injury to roots was not affected.^77^, ^78^ If short‐stature corn is planted at an increased density, this could favor greater corn rootworm adult emergence compared to tall corn with conventional planting density. Additional research to determine which planting densities are tolerated by short‐stature corn, and how these densities might impact corn rootworm management, could help to refine IRM strategies for short‐stature corn.

Due to similarities in patterns of emergence, survival, and root injury observed in this study, current corn rootworm IRM strategies for tall corn may be appropriate for short‐stature corn. However, additional research on patterns of adult emergence, movement, and mating within blended refuges is needed to more fully understand the applicability of blended refuges to delay Bt resistance in short‐stature corn. These studies should address how agronomic changes that may accompany short‐stature corn, such as increased planting density, may influence IRM strategies. The refuge strategy and pyramids likely will continue to be important for delaying Bt resistance in corn rootworm in short‐stature corn. As with tall corn, the use of other pest management options, such as insecticide applications and crop rotation, should be used in rotation with Bt corn to reduce the risk of Bt resistance evolving.^79^

## CONFLICT OF INTEREST

AJG received research funding for this work from Bayer Crop Science and additional research funding, not related to this work, from various agricultural companies including AMVAC, BASF, Bayer, Corteva, FMC, Syngenta, and Valent.

## Data Availability

The data that support the findings of this study are available on request from the corresponding author. The data are not publicly available due to privacy or ethical restrictions.
